# Nicotinamide riboside with pterostilbene (NRPT) increases NAD^+^ in patients with acute kidney injury (AKI): a randomized, double-blind, placebo-controlled, stepwise safety study of escalating doses of NRPT in patients with AKI

**DOI:** 10.1186/s12882-020-02006-1

**Published:** 2020-08-13

**Authors:** Petra Simic, Xavier Fernando Vela Parada, Samir M. Parikh, Ryan Dellinger, Leonard P. Guarente, Eugene P. Rhee

**Affiliations:** 1 Division of Nephrology and Department of Medicine, Massachusetts General Hospital, Harvard Medical School, Boston, MA USA; 2 Division of Nephrology and Department of Medicine, Beth Israel Deaconess Medical Center, Harvard Medical School, Boston, MA USA; 3 Elysium Health Inc., New York, NY USA; 4 Department of Biology, Massachusetts Institute of Technology, Cambridge, MA USA; 5 Broad Institute, Cambridge, MA USA

**Keywords:** Acute kidney injury, NAD^+^, Nicotinamide riboside, Pterostilbene, Safety study

## Abstract

**Background:**

Preclinical studies have identified both NAD^+^ and sirtuin augmentation as potential strategies for the prevention and treatment of AKI. Nicotinamide riboside (NR) is a NAD^+^ precursor vitamin and pterostilbene (PT) is potent sirtuin activator found in blueberries. Here, we tested the effect of combined NR and PT (NRPT) on whole blood NAD^+^ levels and safety parameters in patients with AKI.

**Methods:**

We conducted a randomized, double-blind, placebo-controlled study of escalating doses of NRPT in 24 hospitalized patients with AKI. The study was comprised of four Steps during which NRPT (5 subjects) or placebo (1 subject) was given twice a day for 2 days. NRPT dosing was increased in each Step: Step 1250/50 mg, Step 2500/100 mg, Step 3750/150 mg and Step 41,000/200 mg. Blood NAD^+^ levels were measured by liquid chromatography-mass spectrometry and safety was assessed by history, physical exam, and clinical laboratory testing.

**Results:**

AKI resulted in a 50% reduction in whole blood NAD^+^ levels at 48 h compared to 0 h in patients receiving placebo (*p* = 0.05). There was a trend for increase in NAD^+^ levels in all NRPT Steps individually at 48 h compared to 0 h, but only the change in Step 2 reached statistical significance (47%, *p* = 0.04), and there was considerable interindividual variability in the NAD^+^ response to treatment. Considering all Steps together, NRPT treatment increased NAD^+^ levels by 37% at 48 h compared to 0 h (*p* = 0.002). All safety laboratory tests were unchanged by NRPT treatment, including creatinine, estimated glomerular filtration rate (eGFR), electrolytes, liver function tests, and blood counts. Three of 20 patients receiving NRPT reported minor gastrointestinal side effects.

**Conclusion:**

NRPT increases whole blood NAD^+^ levels in hospitalized patients with AKI. In addition, NRPT up to a dose of 1000 mg/200 mg twice a day for 2 days is safe and well tolerated in these patients. Further studies to assess the potential therapeutic benefit of NRPT in AKI are warranted.

**Trial registration:**

NCT03176628, date of registration June 5th, 2017.

## Background

Acute kidney injury (AKI) is common, growing in incidence, and associated with significant morbidity and mortality. AKI is most commonly diagnosed in patients following major invasive surgical procedures, in the setting of sepsis, or following administration of certain drugs or contrast dyes, particularly in patients with underlying hypertension, diabetes, or chronic kidney disease [1]. There is currently no specific treatment for AKI. Supportive measures include dialysis for patients with severe AKI, and mortality in this subset of patients exceeds 50% [2]. Thus, new preventive and treatment approaches are urgently needed.

Nicotinamide adenine dinucleotide (NAD^+^) is a key cellular factor linked to metabolism and longevity [3]. It is a required co-factor of SIRT1, a nuclear deacetylase that modulates chromatin structure, gene expression, extends lifespan in lower organisms, and improves many aging related diseases [4, 5]. Experimental AKI in mice rapidly leads to the reduction of NAD^+^ levels in the kidney that results from a combination of decreased NAD^+^ biosynthesis and increased NAD^+^ consumption [6]. NAD^+^ augmentation by niacinamide has been shown to prevent various etiologies of experimental AKI in mice, and an initial pilot study has demonstrated the safety of niacinamide in patients undergoing cardiac surgery [7]. Further, mice deficient in SIRT1 are more susceptible to AKI and overexpression of SIRT1 protects from AKI [8]. These studies outline NAD^+^ and SIRT1 modulation as a potential therapeutic approach in AKI [9].

Nicotinamide riboside with pterostilbene (NRPT) is a combination of nicotinamide riboside (NR), a form of vitamin B3, and pterostilbene (PT), a polyphenol found in blueberries [10]. NR is in wide use as an NAD^+^ precursor, with distinct and superior pharmacokinetics to other NAD^+^ intermediates in vitamin B metabolism such as nicotinic acid and niacinamide [11]. Through its effect on NAD^+^ levels and SIRT1 activity, NR has been shown to have metabolic benefits and efficacy in a variety of disease models and to modestly increase lifespan in aged mice [12]. PT is a natural equivalent of the polyphenol resveratrol, an effective SIRT1 activator [13], that has greater bioavailability [14]. Therefore, the combination of NR and PT is predicted to have synergistic effect on metabolism, with NR augmenting NAD^+^ levels and PT additionally activating SIRT1 [10]. NR supplementation alone has been studied in humans with cardiovascular [15], systemic metabolic [16], exercise [17], and muscle aging related end-points [18]. Further, NRPT has been evaluated in healthy elderly adults (age 60 to 80 years) and was shown to safely increase the blood concentration of NAD^+^ in a dose-dependent manner by 40–90% [10]. To date, NR or NRPT have not been tested in patients with kidney disease. Thus, we sought to test the effect of escalating doses of NRPT on blood NAD+ levels and safety parameters in patients hospitalized with AKI, with the ultimate goal to test NRPT for the treatment of AKI.

## Methods

### Study design

**Fig. 1:**
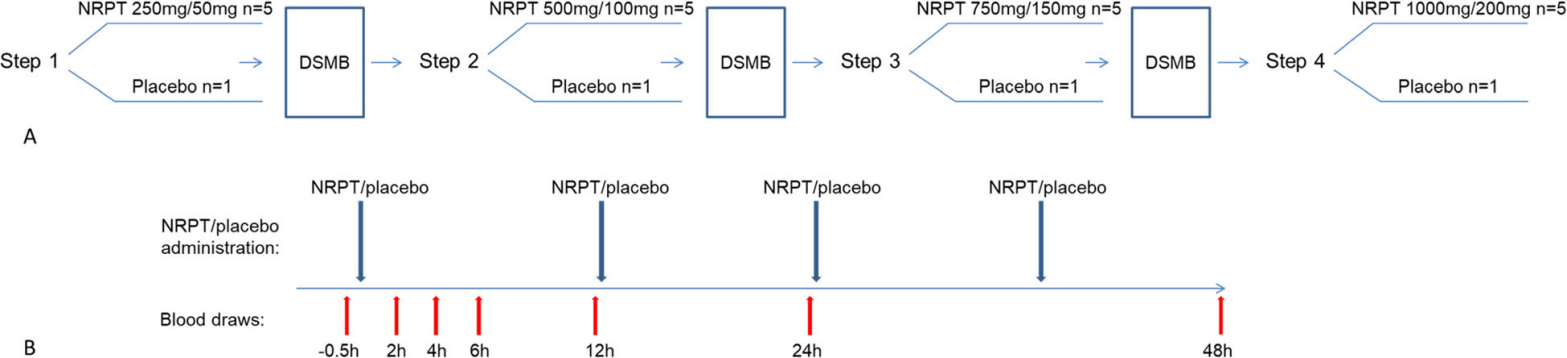
Study Schema. **a** Stepwise approach of administering NRPT or placebo. **b** NRPT administration and blood sampling schedule. NRPT, nicotinamide riboside pterostilbene, DSMB, data safety monitoring board

This was a randomized, double-blind, placebo-controlled, stepwise study of escalating doses of NRPT in hospitalized patients with AKI. This pilot dose-ranging study was comprised of four Steps (Fig. 1a). The purpose of the stepwise approach was to determine the increase in whole blood NAD^+^ levels following NRPT administration, with prespecified secondary goals to determine the dose of NRPT that safely achieves at least a 50% increase, and up to 100% increase, from baseline in whole blood NAD^+^ levels. During each Step, NRPT (5 subjects) or placebo (1 subject) was given twice a day for 2 days (Fig. 1b). Patients had frequent blood sampling for NAD^+^ measurements performed for a 24-h period following the first dose at 0 h and then at 48 h (Fig. 1b). Safety assessment was at 24 h and 48 h after the first dose.

In Step 1 (250 mg/50 mg of NRPT twice a day or placebo), the following schedule was followed: on Day 1 blood was collected 30 min before NRPT or placebo administration and then at 2 h, 4 h, 6 h, 12 h, 24 h, and 48 h. Safety assessment and laboratory tests were performed at 24 h and 48 h after the first dose. After completion of each Step, NAD^+^ levels and safety data were analyzed and used to determine if the study should proceed. In subsequent Steps, doses of active drug were planned at × 2, × 3 or × 4 the Step 1 dose (Fig. 1a).

Safety assessment was performed by patient interviews, vital sign measurements, physical examination, and laboratory tests, including complete blood count and comprehensive metabolic panel. Assessment of adverse events included specific questioning about nausea, abdominal pain, vomiting, diarrhea, and rash. Adverse events were characterized as probably related, probably not related, or unknown and all adverse events were reviewed by a data safety monitoring board (DSMB).

NRPT was supplied by Elysium Inc. in the form of *Basis* and was re-packaged by the Massachusetts General Hospital (MGH) pharmacy in capsules identical to placebo. The MGH pharmacy performed randomization based on the allocation schedule with a 5:1 chance of being assigned to the NRPT group. Both patients and study staff were blinded to the treatment. The study was approved by the MGH Institutional Review Board (IRB) and was registered on ClinicalTrials.gov, identifier NCT03176628. The study was initiated on November 1st, 2017 and completed on September 11th, 2018.

### Study population

Study subjects were selected from hospitalized patients at the MGH.

Inclusion criteria:

1. Male or female hospitalized patient, age ≥ 18 years.
2. Developed AKI during the hospitalization, defined by an increase in serum creatinine by ≥0.3 mg/dL within 48 h; or an increase in serum creatinine to ≥1.5 times baseline. Although urine output totals per 24 h were available for all patients, they were not recorded on an hourly basis as part of routine care, precluding the use of urine output as an inclusion parameter for AKI.
3. Adequate hematological and liver function, as assessed by the following laboratory requirements:
  - Hemoglobin ≥10.0 g/dL
  - Absolute neutrophil count ≥1500/mm^3^
  - Platelet count ≥100,000/mm^3^
  - Total bilirubin ≤1.5 x upper limit of normal
  - ALT and AST ≤2.5 x ULN
4. Able to provide written informed consent in compliance with the Human Investigation Review Committee (IRB).

Exclusion criteria:

1. Exposure to any investigational agent within 30 days prior to enrollment.
2. Known allergy to any of the study drugs or their excipients.
3. Currently pregnant (confirmed with a positive serum pregnancy test) or nursing.
4. Unstable or clinically significant concurrent medical condition, psychiatric illness or social situation that would, in the opinion of the investigator, jeopardize the safety of a subject and/or their compliance with the protocol.
5. Baseline chronic kidney disease (CKD) stage 4–5 (estimated glomerular filtration rate, eGFR< 30 mL/minute/1.73 m^2^ as determined using the Modification of Diet in Renal Disease equation) prior to current hospitalization.
6. Any malignancy with the exception of cervical carcinoma in situ, nonmelanoma skin cancer, or superficial bladder tumors that have been successfully and curatively treated with no evidence of recurrent or residual disease.

Eligible subjects were identified through the electronic medical record. Patients were not approached until permission was given by their treating physician. Subjects had greater than 12 h to review the consent prior to agreeing to participate in the study. The cause of AKI was identified by the primary team and was verified by two independent investigators. The stage of AKI was determined using Acute Kidney Injury Network (AKIN) serum creatinine criteria: stage 1, 150–200%; stage 2, 200–300%; stage 3 > 300% increase from baseline. As AKI developed during the hospitalization for all participants, baseline creatinine was defined as the last creatinine value prior to AKI, i.e. during the same hospitalization. In addition, we recorded the most recent creatinine value available in the electronic medical record prior to the current admission.

### NAD^+^ measurement

NAD^+^ measurement was performed from blood samples as previously described [10]. In brief, 4 mL of blood was collected and 0.1 mL aliquots were then transferred to cryovials containing 1 mL of 0.5 M perchloric acid (PCA), used for lysis of blood cells. NAD^+^ was measured by liquid chromatography-mass spectrometry (LC-MS) by Keystone Bioanalytical. In brief, samples were centrifuged and 0.11 mL of supernatant was mixed with 100 μL of 0.5 M PCA in water in HPLC vial. Fifty microliters of 5 μg/mL of ^13^C5-nicotinamide adenine dinucleotide in 0.5 M PCA (internal standard solution) and 0.5 mL of 0.5 M PCA in water were added. Ten microliters were injected onto the LC-MS. NAD^+^ was quantitated based on d5-NAD^+^ as an internal standard. 0.5% formic acid in water was mobile phase A and 0.5% formic acid in acetonitrile was mobile phase B. The mass spectrometer was set to monitor for the transitions of 664.4 → 524.0 (NAD^+^) and 669.4 → 529.3 (internal standard) in a positive ion mode.

### Data safety monitoring board (DSMB)

An independent DSMB comprised of 3 physicians reviewed efficacy data (NAD^+^ levels) and safety data results at the completion of each Step of the protocol. The investigators assessed and recorded any adverse event in detail including the date of onset, description, severity, time course, duration and outcome, relationship of the adverse event to study drug, an event diagnosis, if known, and any action(s) taken. All adverse events were reported to the DSMB and to the manufacturer of *Basis*.

### Study analyses

All randomized patients were included in intention-to-treat analyses. NAD^+^ data were analyzed descriptively and graphically. Safety endpoints were assessed by patient interview and physical examination conducted by a study physician, along with review of vital signs and laboratory tests (complete blood count and comprehensive metabolic panel). Data from different Steps were analyzed separately and collectively for Steps 1–4 vs 0 h (baseline) and vs placebo. Adverse events were analyzed descriptively and characterized as probably related, probably not related, or unknown. Data are expressed as analyte or laboratory value mean ± standard deviation in the Tables and mean ± standard error of the mean in the Figures. The study adheres to CONSORT guidelines. Statistical comparison of means is performed by two-tailed unpaired Student’s t test or ANOVA depending on the number of groups analyzed. The null hypothesis was rejected at *p* < 0.05.

## Results

### Study population

**Fig. 2:**
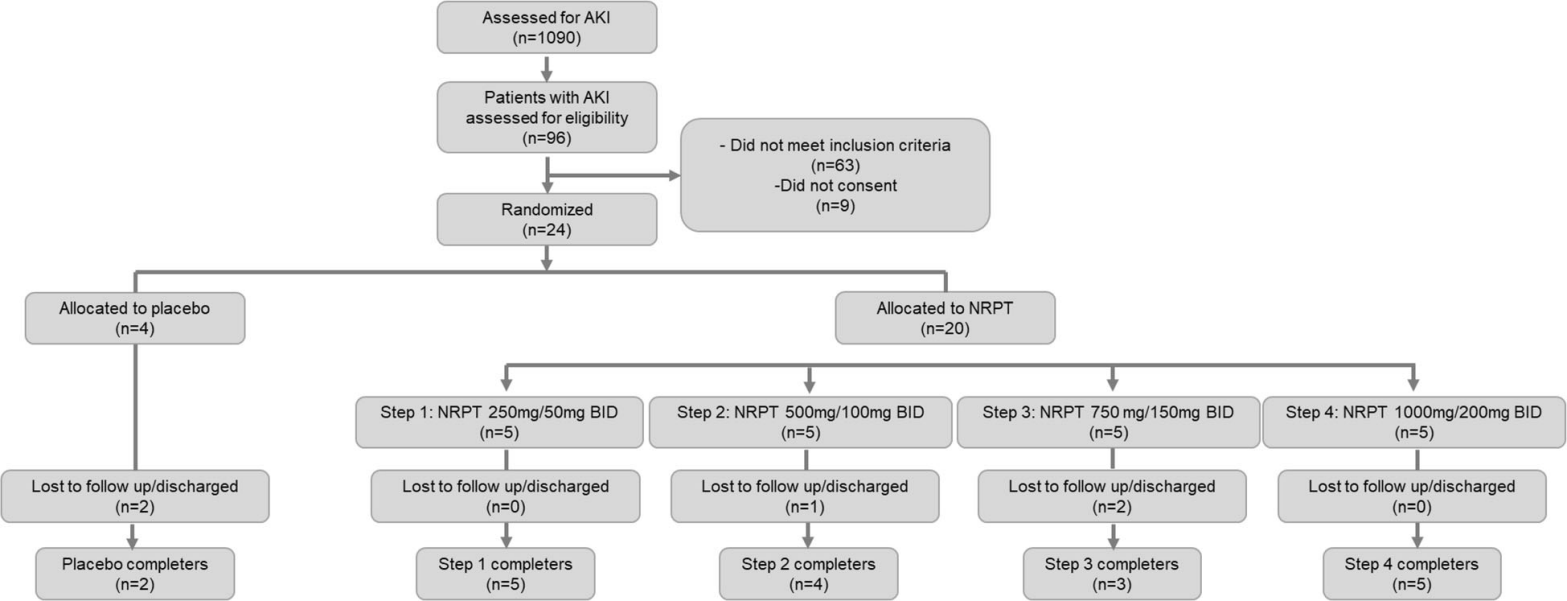
Study Flow Diagram. NRPT, nicotinamide riboside pterostilbene

Of 1090 hospitalized patients that were screened, 96 (8.8%) had AKI and 33 (3%) of these patients with AKI met the study’s inclusion criteria. Sixty-three patients with AKI were excluded because of malignancy, an increase in liver enzymes or total bilirubin, or decrease in platelets. Of the 33 patients who met the inclusion criteria, 24 agreed to participate in the study across a total of four Steps (Fig. 2). A total of 4 patients were randomized to placebo and 20 patients were randomized to NRPT. Of all patients randomized, 2 participants in the placebo group (50%) and 17 participants receiving NRPT (85%) completed the study. Two patients in the placebo group, 1 patient receiving NRPT in Step 2 and 2 patients receiving NRPT in Step 3 were discharged from the hospital prior to completion of the study. The study was performed between January 9th, 2018 until July 21st, 2018.

Demographic and baseline characteristics of the study subjects are shown in Table 1. For the placebo group, mean age was 74 years, 1 participant (25%) was male, and all participants (100%) were White. For the NRPT group, mean age was 68.3 years, 16 participants (80%) were male, and 17 participants (85%) were White. Mean baseline creatinine was similar in both groups (1.29 ± 0.25 mg/dl in the placebo group and 1.30 ± 0.29 mg/dl in the NRPT group), with no significant difference in eGFR between groups (Table 1). The most recent creatinine prior to the current hospitalization available in the electronic medical record was also similar between the groups (mean 1.27 ± 0.18 mg/dl in the placebo group and 1.30 ± 0.33 mg/dl in the NRPT group) and was recorded on average 37 days prior to AKI in the placebo group and 93 days prior to AKI in the NRPT group. The most common cause of AKI was drug induced (e.g. antibiotics or diuretics), responsible for AKI in 2 participants (50%) in the placebo group and 8 participants (40%) in the NRPT group, followed by cardiorenal syndrome, iodinated contrast for imaging, and hypotension (Table 1). Mean duration of AKI prior to intervention was 2 days in the placebo group and 1.8 days in the NRPT group (Table 1) with serum creatinine assessed daily. The majority of patients (23/24) had stage 1 AKI and one patient in the NRPT 500/100 mg treatment group had stage 2 AKI. There was no difference in the mean daily urine output (1.66 L/day in the placebo group and 1.74 L/day in the NRPT group).

**Table 1:** Study Demographics

	Placebo ***n*** = 4	Total NRPT ***n*** = 20	NRPT Dose			
			250/50 mg ***n*** = 5	500/100 mg ***n*** = 5	750/150 mg ***n*** = 5	1000/200 mg ***n*** = 5
Age (years)						
Mean ± SD	74.0 ± 4.3	68.3 ± 12.8	67.9 ± 7.2	70.4 ± 8.2	62.2 ± 17.3	73.9 ± 6.7
Sex, *n* (%)						
Male	1 (25)	16 (80)	5 (100)	3 (60)	4 (80)	4 (80)
Race, *n* (%)						
White	4 (100)	17 (85)	4 (80)	4 (80)	4 (80)	5 (100)
African American	0	1 (5)	0	1 (20)	0	0
Asian	0	2 (10)	1 (20)	0	1 (20)	0
Other	0	0	0	0	0	0
Baseline kidney function, Mean ± SD						
Cr (mg/dl)	1.29 ± 0.25	1.30 ± 0.29	1.37 ± 0.28	1.27 ± 0.16	1.29 ± 0.47	1.26 ± 0.26
eGFR (ml/min/1.73m^2^)	47.0 ± 15.4	56.7 ± 14.6	52.4 ± 10.5	54.0 ± 12.9	60.0 ± 19.3	58.6 ± 15.9
BUN (mg/dl)	49.5 ± 23.0	26.45 ± 11.25	31.2 ± 16.5	23.6 ± 5.9	20.8 ± 9.3	30.2 ± 13.3
Cause of AKI, *n* (%)						
Drug induced	2 (50)	8 (40)	2 (40)	1 (20)	2 (40)	3 (60)
Cardiorenal	2 (50)	7 (35)	2 (40)	2 (40)	2 (40)	1 (20)
Contrast		3 (15)	1 (20)	2 (40)		
Hypotension		2 (10)			1 (20)	1 (20)
Characteristics of AKI prior to intervention, Mean ± SD						
Days of AKI	2.0 ± 0.8	1.8 ± 0.7	1.6 ± 0.5	2.0 ± 0.7	1.6 ± 0.5	2.0 ± 1.0
AKIN stage (n)	1 (4)	1 (19), 2 (1)	1 (5)	1 (4), 2 (1)	1 (5)	1 (5)
UOP (L/day)	1.66 ± 0.91	1.74 ± 0.90	2.00 ± 1.39	1.35 ± 0.80	1.67 ± 0.42	1.94 ± 0.90

*Cr* creatinine, *eGFR* estimated glomerular filtration rate, *BUN* blood urea nitrogen, *AKIN* acute kidney injury network, *UOP* urine output

### The effect of NRPT on whole blood NAD^+^ levels in patients with AKI

**Fig. 3:**
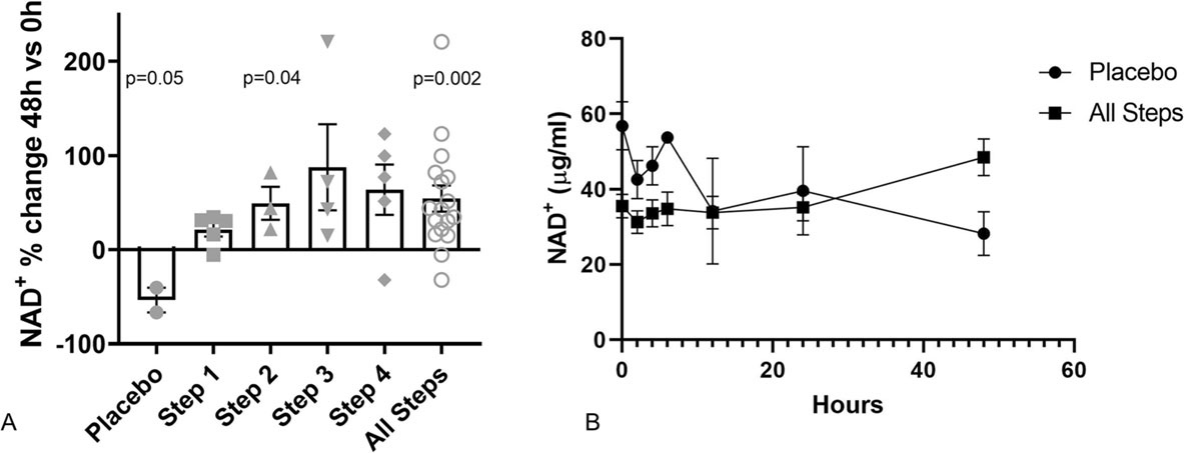
Whole blood NAD^+^ levels in patients with AKI treated with NRPT or placebo. **a** Percent change in whole blood NAD^+^ levels at 48 h as compared to 0 h. **b** Whole blood NAD^+^ levels in all Steps combined as compared to placebo. Data is shown as mean and SEM

Compared to the baseline timepoint, NRPT at all doses increased whole blood NAD^+^ levels at 48 h (Fig. 3a), but only the increase with NRPT Step 2 (dose 500 mg/100 mg) reached statistical significance (47%, *p* = 0.04). The highest mean increase in NAD^+^ was achieved in Step 3 (67%, dose 750 mg/150 mg), but this increase was not statistically significant. As shown, the change in whole blood NAD^+^ levels following NRPT administration demonstrated significant inter-subject variability. When all NPRT Steps were combined, NRPT treatment demonstrated a significant increase in whole blood NAD^+^ levels at 48 h compared to baseline (37%, *p* = 0.002). No significant change in whole blood NAD^+^ levels was noted following NRPT treatment at earlier time points. Notably, whole blood NAD^+^ levels in patients with AKI receiving placebo decreased by 50% at 48 h (*p* = 0.05, Fig. 3a). As a result, NPRT treatment (all Steps combined) demonstrated an 80% increase in NAD^+^ levels compared to placebo at 48 h (Fig. 3b), although this difference was not statistically significant (*p* = 0.19).

### Safety of NRPT in patients with AKI

**Fig. 4:**
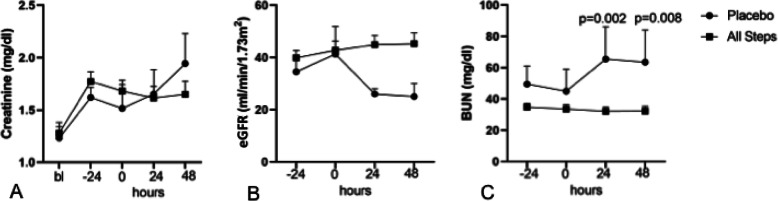
Renal Function Safety Laboratory Tests. **a** Creatinine; **b** estimated glomerular filtration rate (eGFR); and **c** blood urea nitrogen (BUN) in patients treated with all doses of NRPT combined (all Steps) or placebo. Bl, baseline, prior to AKI; −24 h, at the time of enrollment; 0 h, immediately prior to first dose of NRPT or placebo; 24 h, at 24 h of treatment; 48 h, at 48 h of treatment

NRPT was safe in all Steps in patients with AKI. Compared to placebo, there was no significant difference in creatinine and eGFR in patients treated with NRPT at 48 h (Fig. 4a, b). Blood urea nitrogen (BUN) was lower at 48 h in patients treated with NRPT compared to placebo (Fig. 4c). There was no difference in electrolytes (sodium, potassium), liver enzymes (ALT, AST, ALP), hemoglobin, white blood count or platelet count between placebo and NRPT treated groups at 48 h (Table 2).

**Table 2:** Safety Laboratory Tests

	Placebo		Step1		Step2		Step3		Step 4	
	0 h	48 h	0 h	48 h	0 h	48 h	0 h	48 h	0 h	48 h
Cr (mg/dl)	1.52 ± 0.45	1.95 ± 0.40	1.66 ± 0.27	1.62 ± 0.33	1.62 ± 0.48	1.56 ± 0.64	1.9 ± 0.69	1.79 ± 0.84	1.54 ± 0.41	1.62 ± 0.51
eGFR (ml/min/1.73m^2^)	41.3 ± 21.1	25.0 ± 7.1	43.2 ± 9.9	47.8 ± 11.5	42.0 ± 20.4	50.0 ± 28.1	39.2 ± 13.3	41.3 ± 15.7	46.4 ± 21.8	43.6 ± 17.5
BUN (mg/dl)	45.0 ± 28.1	63.5 ± 29.0	36.8 ± 15.6	34.6 ± 12.1	33.5 ± 9.3	28.0 ± 13.1	35.6 ± 12.6	37.3 ± 9.3	28.4 ± 12.0	28.6 ± 16.2
Na (mEq/l)	140.0 ± 2.2	138.5 ± 0.7	140.6 ± 3.5	138.8 ± 5.1	140.0 ± 2.9	138.3 ± 2.5	140.6 ± 3.6	140.0 ± 2.9	137.2 ± 3.3	136.2 ± 2.6
K (mEq/l)	4.1 ± 0.4	4.0 ± 0.1	4.2 ± 0.5	4.6 ± 0.9	4.3 ± 0.4	4.1 ± 0.2	4.2 ± 0.5	4.5 ± 0.5	4.3 ± 0.5	4.3 ± 0.5
AST (U/l)	20.0 ± 6.6	19.5 ± 2.1	25.0 ± 9.8	60.5 ± 55.9	33.8 ± 12.7	33.0 ± 2.8	27.2 ± 8.6	27.7 ± 17.8	25.6 ± 13.3	24.0 ± 1.9
ALT (U/l)	14.5 ± 3.9	13.0 ± 1.4	24.5 ± 7.1	103.0 ± 117.4	15.8 ± 3.1	18.8 ± 5.7	20.2 ± 12.1	23.0 ± 15.7	15.6 ± 7.5	18.0 ± 6.7
ALP (U/l)	109.8 ± 47.2	105.0 ± 33.9	116.3 ± 64.4	143.5 ± 62.9	148.0 ± 120.2	117.3 ± 65.1	86.3 ± 39.7	91.3 ± 38.5	79.8 ± 29.3	111.6 ± 37.3
WBC (K/μl)	6.9 ± 3.4	8.5 ± 1.4	8.9 ± 2.1	9.0 ± 1.8	10.1 ± 2.9	9.2 ± 2.0	9.3 ± 2.8	8.2 ± 2.3	10.2 ± 5.7	8.8 ± 2.5
Hgb (g/dl)	11.0 ± 2.0	9.9 ± 3.1	10.9 ± 2.9	11.4 ± 3.0	11.1 ± 3.3	10.8 ± 3.3	10.3 ± 1.7	10.7 ± 1.5	11.3 ± 2.0	11.1 ± 2.4
PLT (K/μl)	207.3 ± 37.3	225.5 ± 17.7	202.8 ± 68.7	240.0 ± 28.1	260.5 ± 74.5	234.3 ± 59.4	286.0 ± 136.7	238.0 ± 74.7	268.3 ± 75.6	269.0 ± 108.4

*Cr* creatinine, *eGFR* estimated glomerular filtration rate, *BUN* blood urea nitrogen, *Na* sodium, *K* potassium, *AST* aspartate aminotransferase, *ALT* alanine aminotransferase, *ALP* alkaline phosphatase, *WBC* white blood cells, *Hgb* hemoglobin, *PLT* platelets; Data are expressed as laboratory value mean ± standard deviation

### Adverse events

NRPT was safe at all doses with only minor side effects reported that resolved without intervention (Table 3). One patient from Step 1 complained of bloating and gas, while two patients in Step 2 had indigestion with upper abdominal discomfort. There were no side effects reported in placebo, Step 3, and Step 4 groups and there were no serious adverse events.

**Table 3:** Adverse Events

	Placebo	All Steps	Step 1	Step 2	Step 3	Step 4
AE	0/4	3/20	1/5 (gas)	2/5 (indigestion)	0/5	0/5
SAE	0/4	0/20	0/5	0/5	0/5	0/5

*AE* adverse event, *SAE* serious adverse events

## Discussion

The incidence of AKI in hospitalized patients is about 1.6% with mortality rate of up to 35% [19]. AKI is associated with higher rates of death, subsequent hospitalization for stroke, heart failure, or myocardial infarction [20] and it accelerates the progression of CKD [21]. The management of patients with AKI is supportive, with renal replacement therapy indicated in patients with severe kidney injury. Currently, there are no specific therapeutics for the prevention and or management of AKI. Most studies in patients with AKI have focused on AKI after cardiac surgery, investigating renal perfusion, including studies investigating dopamine and neseritide, fenoldopam, aspirin or clonidine, buffered crystalloid, and remote ischemic preconditioning (reviewed in [22]), all to no avail. Trials examining the use of sodium bicarbonate or N-acetylcysteine in contrast induced nephropathy prevention have been inconclusive [23]. Because this prior focus on hemodynamics and perfusion has been unsuccessful, there has been increasing interest in augmenting kidney metabolic health by increasing NAD^+^ levels and action as a novel therapeutic approach in AKI [7, 9].

To our knowledge this is the first study describing whole blood NAD^+^ levels in human AKI, with or without intervention. Across all treated individuals, NRPT treatment resulted in a significant increase in whole blood NAD^+^ at 48 h. In placebo treated individuals, AKI resulted in a 50% decrease in whole blood NAD^+^ levels at this time point. NRPT at all four tested doses was safe and well tolerated. These results motivate additional studies of NRPT as a potential therapeutic option in AKI.

Our prespecified secondary endpoint was to determine the dose of NRPT that safely achieves at least a 50% increase (and up to a 100% increase) from baseline in cellular NAD^+^ levels. The only dose of NRPT that resulted in a statistically significant increase in whole blood NAD^+^ levels at 48 h as compared to 0 h was 500 mg/100 mg in Step 2, yielding a 47% increase. The Step 3 dose resulted in a 67% increase in whole blood NAD^+^ levels that was not statistically significant. Thus in retrospect, the initial goal of a 50 to 100% increase in NAD^+^ levels was likely set too high, in large part because it was based on studies in healthy volunteers [10, 11] without accounting for such a profound decrease in whole blood NAD^+^ levels with kidney injury (50% decrease after 2 days of AKI).

NR alone was previously tested at similar doses (100, 300 and 1000 mg) in a study of 12 healthy volunteers, resulting in a significant increase in peripheral blood mononuclear cell NAD^+^ levels after 24 h (all NR doses combined) and no effect during earlier time points (1, 2, 4 and 8 h) [11]. The relative increase in NAD^+^ at 24 h vs 0 h was 46% for both 300 mg and 1000 mg of NR and only 12% for 100 mg of NR. NAD^+^ was not measured beyond 24 h in this study [11]. We now show, in patients with AKI, a comparable increase in NAD^+^ levels (40% at 48 h vs 0 h in all Steps combined), but with delayed effect to 48 h. This could represent slower incline in cellular NAD^+^ in patients with AKI or the effect of PT. We have previously tested the combination of NR and PT (NRPT 250 mg/50 mg and 500 mg/100 mg) in 120 elderly healthy volunteers, with the focus on NAD^+^ levels at later time points, namely after 30 and 60 days of treatment [10]. After 30 days of treatment, whole blood NAD^+^ levels in the healthy elderly population increased by 40% in NRPT 250/50 mg and 90% in NRPT 500/100 mg treatment groups and the effect was sustained until 60 days [10]. In our study, NRPT at the same doses in an AKI population increased whole blood NAD^+^ levels by 23 and 47% respectively at 48 h. It is possible that the increase in NAD^+^ values would have been greater with more prolonged dosing and at later time points.

Reduced kidney function often leads to drug toxicity because almost half of all medications used are eliminated via the kidney [24]. NRPT was generally well tolerated and raised no safety concerns at doses up to 1000 mg of NR and 200 mg of PT twice a day. Furthermore, the gastrointestinal adverse events observed (bloating and indigestion) might not even be related to NRPT as the study was comprised of hospitalized patients receiving multiple other medications. All safety laboratory tests remained unchanged with the exception of BUN that was decreased after the treatment with NRPT.

Several limitations warrant mention. First, inherent in a pilot, dose-ranging trial, this study was of modest sample size and treatment duration. There was no control of timing of discharge from the hospital, so two out of four placebo patients were discharged prior to completion of the study, limiting the power of our comparisons of treatment versus placebo. Second, the study focused on individuals who could provide written informed consent after the onset of AKI. As a result, we enrolled subjects with mild AKI due to a range of non-life threatening etiologies. We acknowledge that the impact of NRPT on blood NAD^+^ levels could be further impacted by dietary or inflammatory factors that accompany AKI in more acute settings, as with major cardiac surgery or sepsis. Additionally, because of our enrollment strategy, NRPT was administered relatively late during AKI; after being identified as having an increase in serum creatinine within 48 h, patients had an additional day prior to starting treatment to allow for the consent process, randomization and study drug blinding by pharmacy. Third, NAD^+^ was measured in whole blood, which may not accurately reflect kidney tissue NAD^+^ levels, and PT was not measured.

## Conclusion

NRPT increases whole blood NAD^+^ levels among patients with mild AKI. However, there is considerable interindividual variability in the NAD^+^ response to NRPT and the increase occurs at 48 h, suggesting strategies with earlier administration should be considered in future studies. NRPT is safe and well tolerated at all doses in patients with AKI. Further phase II studies are needed to evaluate the efficacy of NRPT on renal outcomes, including individuals with more severe injury and a broader range of AKI etiologies.

## Data Availability

The datasets analyzed during the current study are available from the corresponding author on reasonable request.

## Abbreviations

AKI: Acute kidney injury
BUN: Blood urea nitrogen
CKD: Chronic kidney disease
Cr: Creatinine
DSMB: Data safety monitoring board
eGFR: Estimated glomerular filtration rate
IRB: Institutional Review Board
LC-MS: Liquid chromatography-mass spectrometry
MGH: Massachusetts General Hospital
NAD^+^: Nicotinamide adenine dinucleotide
NR: Nicotinamide riboside
NRPT: Nicotinamide riboside with pterositlbene
PCA: Perchloric acid
PT: Pterostilbene
